# Identification of TNF-α and MMP-9 as potential baseline predictive serum markers of sunitinib activity in patients with renal cell carcinoma using a human cytokine array

**DOI:** 10.1038/sj.bjc.6605409

**Published:** 2009-11-10

**Authors:** J L Perez-Gracia, C Prior, F Guillén-Grima, V Segura, A Gonzalez, A Panizo, I Melero, E Grande-Pulido, A Gurpide, I Gil-Bazo, A Calvo

**Affiliations:** 1 Department of Medical Oncology, University Clinic of Navarra, University of Navarra, Pamplona, Spain; 2 Division of Oncology, CIMA, University of Navarra, Pamplona, Spain; 3 Department of Preventive Medicine, University Clinic of Navarra, University of Navarra, Pamplona, Spain; 4 Genomics, Proteomics and Bioinformatics Unit, CIMA. University of Navarra, Pamplona, Spain; 5 Department of Biochemistry, University Clinic of Navarra, University of Navarra, Pamplona, Spain; 6 Department of Pathology, University Clinic of Navarra, University of Navarra, Pamplona, Spain; 7 Department of Internal Medicine, University Clinic of Navarra and CIMA, University of Navarra, Pamplona, Spain; 8 Department of Medical Oncology, Pfizer Inc., Madrid, Spain

**Keywords:** sunitinib, renal cell carcinoma, TNF-α, MMP-9, selection of extreme phenotypes

## Abstract

**Background:**

Several drugs are available to treat metastatic renal-cell carcinoma (MRCC), and predictive markers to identify the most adequate treatment for each patient are needed. Our objective was to identify potential predictive markers of sunitinib activity in MRCC.

**Methods:**

We collected sequential serum samples from 31 patients treated with sunitinib. Sera of six patients with extreme phenotypes of either marked responses or clear progressions were analysed with a Human Cytokine Array which evaluates 174 cytokines before and after treatment. Variations in cytokine signal intensity were compared between both groups and the most relevant cytokines were assessed by ELISA in all the patients.

**Results:**

Twenty-seven of the 174 cytokines varied significantly between both groups. Five of them (TNF-α, MMP-9, ICAM-1, BDNF and SDF-1) were assessed by ELISA in 21 evaluable patients. TNF-α and MMP-9 baseline levels were significantly increased in non-responders and significantly associated with reduced overall survival and time-to-progression, respectively. The area under the ROC curves for TNF-α and MMP-9 as predictive markers of sunitinib activity were 0.83 and 0.77.

**Conclusion:**

Baseline levels of TNF-α and MMP-9 warrant further study as predictive markers of sunitinib activity in MRCC. Selection of patients with extreme phenotypes seems a valid method to identify potential predictive factors of response.

Treatment of metastatic renal cell carcinoma (MRCC) has rapidly evolved during the past years, with the development of several new targeted agents. Sunitinib (Sutent; Pfizer Inc., New York, NY, USA) and the combination of bevacizumab and interferon have shown superiority over single-agent interferon in randomized phase III trials as first-line treatment of MRCC (Motzer *et al*, 2007; Escudier *et al*, 2007b; Rini *et al*, 2008a). Temsirolimus was also superior to interferon in patients with poor prognosis MRCC (Hudes *et al*, 2007). Finally, sorafenib and everolimus have also shown efficacy in the second-line setting (Escudier *et al*, 2007a; Motzer *et al*, 2008b). Even though clinical algorithms that recommend the most appropriate agent according to each clinical situation and previous treatments have been developed (Motzer *et al*, 2008c), predictive markers of activity to determine the most appropriate treatment for each patient are urgently needed to maximise the clinical benefit and to spare unnecessary toxicities and costs.

Selection of predictive markers of response is an intricate task, given the large number of potential targets to analyse. High-throughput techniques have the advantage of being able to evaluate several molecular factors at one time, but their interpretation and their translation into clinical practice is somewhat cumbersome. The selection and study of individuals with very characteristic and clinically relevant phenotypes has been proposed as a methodology that may help to characterize the factors underlying such phenotypes (Perez-Gracia *et al*, 2002). This strategy consists of selecting and screening with high-throughput techniques of those patients presenting the most informative clinical features, rather than whole unselected series of patients, to improve the probability of finding factors that might be linked with such phenotypes. We employed this methodology to identify potential predictive factors of response to sunitinib in MRCC.

## Materials and methods

From November 2004 to October 2006, we obtained sequential serum samples from 31 consecutive patients with MRCC treated with sunitinib at our institution, while participating in the phase III sunitinib registration study (Motzer *et al*, 2007) and in an expanded use trial. Patients received sunitinib (50 mg per 24 h) during 4 weeks followed by a 2-week resting period between cycles. Follow-up and toxicity management were performed following the study protocol. Response was evaluated every two cycles using RECIST criteria (Therasse *et al*, 2000). Blood samples were obtained from patients at baseline and at the moment of first evaluation of response at the end of the second cycle (day 28). Serum was obtained after blood centrifugation at 1500 r.p.m. for 10 min at 4°C. Samples were aliquoted and stored at −80°C. Both trials, as well as the protocol to obtain blood samples were approved by our institution's Ethics Committee, and all patients signed the respective informed consents.

The methodology of extreme phenotype selection was used for this study (Perez-Gracia *et al*, 2002). Three patients with very marked clinical responses to sunitinib and three with clear progressions, despite adequate performance status, absence of comorbidities and correct treatment administration, were selected by the attending clinicians. Sera were analysed with a cytokine array that is described below. The most relevant candidate cytokines, in terms of statistical significance and biological plausibility, were selected and they were assessed in the rest of the patients using ELISA.

### High-throughput serum cytokine assay

In a first set of experiments we sought to identify serum cytokines that were modulated by sunitinib in MRCC patients. A Human Cytokine Antibody Array (Series 2000, RayBiotech, Norcross, GA, USA), which has been clinically validated (Ray *et al*, 2007), was used to analyse 174 human cytokines related to angiogenesis, immunity and tumour proliferation pathways (the complete list of cytokines is available as Supplementary Material, Appendix 1). Sera of three patients with an excellent response to sunitinib and of three non-responders were analysed with the array. The baseline cytokine array profile was compared with that found after treatment for each patient. Samples were analysed according to the manufacturer's instructions. Briefly, wells of the microarray glass slides were blocked in blocking buffer for 30 min and subsequently incubated with 1 : 5 diluted samples (90 μl per well) overnight at 4°C. Slides were washed in washing buffer and incubated with a biotin-conjugated cocktail of antibodies for 2 h. After further washing, horseradish peroxidase (HRP)-conjugated streptavidin was incubated for 2 h. To visualise bound antibodies, samples were incubated with Alexa flour 555-conjugated streptavidin in darkness for 2 h. Detection of signals was assessed with a GMS 418 array scanner (Genetic Microsystems, Woburn, MA, USA). Spots signal intensities were imported into a GMS array scanner programme for analysis.

### Array data normalisation and analysis

Positive control signals on each array were used as normalization factor following the manufacturer's instructions. For each cytokine, baseline intensity levels were compared with those found after treatment and fold-change differences were calculated. Global loess-based normalization was performed using Bioconductor (Gentleman *et al*, 2004) and ratios were scaled based on the median absolute deviation. Filtering of flat profiles was applied using a variation coefficient value of 0.5. Statistical comparison of the two experimental conditions was performed using the Wilcoxon test, and the obtained *P*-value was corrected for multiple hypotheses testing using the False Discovery Rate (FDR) method for array analysis. The FDR of a test is defined as the expected proportion of false positives among the declared significant results (Benjamini and Hochberg, 1995, 2000; Keselman *et al*, 2002). The selection of cytokines for ELISA validation was based on an FDR threshold of 10% (Storey and Tibshirani, 2003).

### ELISA

Serum samples obtained at baseline and at the moment of response evaluation were assayed by ELISA (RayBiotech), following the manufacturer's protocol, for TNF-α, MMP-9, ICAM-1, BDNF, VEGF, and SDF-1. Briefly, 96-well plates were incubated at 4°C overnight with standards at different concentrations, and serum samples. After several washes, wells were incubated with biotinylated antibodies for 1 h, followed by HRP-conjugated streptavidin for 45 min. Enzymatic reactions were developed and the absorbance was measured at 450 nm (A_450_) in a SunRise (Tecan, Salzburg, Austria) ELISA plate reader. Protein levels were calculated according to standard curves.

### Statistical methods

Array data was analysed as described above. Statistical differences between groups were examined using the Student's *t*-test for unpaired and paired data for parametric variables, and the Mann–Whitney *U*-test for unpaired non-parametric variables. The Wilcoxon test was used to study paired non-parametric variables. Normality was tested using Shapiro–Wilks Test. Data were analysed with the SPSS statistical software (version 15.0 for Windows SPSS). *P*-values <0.05 were considered significant and 95% confidence intervals were calculated. Time-to-event variables were assayed with the Kaplan–Meier test. Survival curves were compared with the Log-rank test. Risk of lack of response was measured by exact logistic regression (pack logXact V.8). Sensitivity, specificity, predictive values, and receiver operator characteristic (ROC) curves, as well as their confidence intervals were computed with Epitable from Epiinfo v6.1. Discriminant function analysis and Cox regression were performed with SPSS.

## Results

### Variations in serum levels of 27 cytokines from the array showed significant differences between responders and non-responders

**Figure 1:**
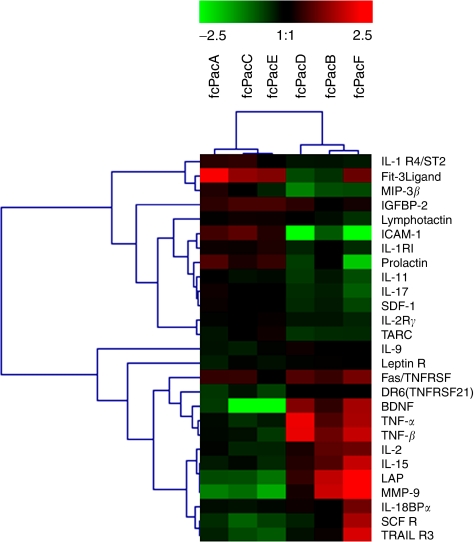
Cluster analysis of antibody-based cytokine microarray in patients treated with sunitinib. Fold-change between baseline levels and levels at the time of evaluation of response were analysed in three patients with response (left columns) and three patients with progression (right columns). The analysis shows that fold-change levels of 27 cytokines cluster responders from non-responders into two different groups.

Twenty-seven of the 174 cytokines assayed by the array were able to cluster the patients in two different groups and to discriminate those with marked clinical responses (*n*=3) from those with clear disease progression (*n*=3), according to the FDR method for array analysis, using a threshold of 10% (Figure 1). Levels of cytokines such as matrix metalloproteinase-9 (MMP-9), tumour necrosis factor-α (TNF-α) and brain-derived neurotrophic factor (BDNF) increased exclusively in non-responding patients, as compared with responders. Conversely, other soluble proteins such as intercellular adhesion molecule-1 (ICAM-1), macrophage inflammatory protein 3β, stromal cell-derived factor-1 (SDF-1) and interleukin-17 decreased significantly only in non-responders and either did not change or were increased in responders.

### Baseline levels of MMP-9 and TNF-α predict clinical benefit of sunitinib

From the 27 cytokines modulated by sunitinib identified by the cytokine array, five (TNF-α, MMP-9, ICAM-1, BDNF, and SDF-1) were selected for further quantitative analysis by ELISA in the whole patient series, based on statistical significance and on biological plausibility of the involvement of such cytokines in renal cell carcinoma. TNF-α was selected because recent reports have demonstrated its involvement in renal carcinoma development, and targeted therapies using anti-TNF-α have been recently shown to be efficacious against this tumour (Harrison *et al*, 2007). MMP-9, whose levels are significantly high in MRCC patients, induces VEGF availability (Hawinkels *et al*, 2008). BDNF upregulates MMP-9 levels and promotes its activation (Sun *et al*, 2006) and, therefore, was also considered for validation by ELISA. SDF-1 was suggested by previous studies as a target for renal carcinoma therapy (Reckamp *et al*, 2008), and increased levels of this cytokine have been shown in sunitinib-treated mice (Ebos *et al*, 2007). ICAM-1 levels were also checked for validation, because plasma levels of this cytokine have been associated with a response to the anti-VEGF targeting agent bevacizumab in combination with docetaxel in metastatic breast cancer (Ramaswamy *et al*, 2006). Vascular endothelial growth factor (VEGF) was quantified as well, because its receptors are primary targets of sunitinib.

Clinical characteristics of the patients are summarised in Table 1. All the patients presented metastatic renal clear-cell carcinoma. The analysis was performed in 21 evaluable patients. Ten patients were not evaluable, due to lack of follow-up information (three patients) or adequate sequential serum samples (seven patients). Twelve patients showed clinical benefit (response or disease stabilisation) and nine patients presented tumour progression.

**Figure 2:**
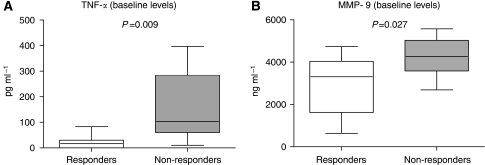
Determination by ELISA of serum baseline levels of TNF-α (**A**) and MMP-9 (**B**) in MRCC patients treated with sunitinib. Inbox bars show median levels for each cytokine of each group of patients. (TNF-α: 20pg ml^−1^ for responders, and 103.3 pg ml^−1^ for non-responders; MMP-9: 3303.9 ng ml^−1^ for responders, and 4262.4 ng ml^−1^ for non-responders). A significant increase in non-responders compared with responders is found.

Baseline levels of TNF-α and MMP-9 were significantly higher (*P*<0.05) in patients who progressed (Figure 2, Table 2) than in patients with no progression, whereas no differences were seen for the rest of the cytokines (Table 2). To measure the accuracy of the MMP-9 and TNF-α ELISA tests to predict a lack of response to sunitinib, sensitivity (S), specificity (Sp), positive (PPV) and negative (NPV) predictive values, and area under the ROC curves were calculated. For MMP-9, using the upper tercile (4062 ng ml^−1^) as a cutoff value, data were as follows: S: 55.6% (95% CI: 22.7–84.7); Sp: 75% (95% CI: 42.8–93.3); PPV: 62.5 (95% CI: 25.9–89.8); NPV: 69.2 (95% CI: 38.9–89.6). For TNF-α, using the median (50 pg ml^−1^) as a cutoff value, the following results were obtained: S: 88% (95% CI: 50.7–99.4); Sp: 75% (95% CI: 42.8–93.3); PPV: 72.7% (95% CI: 39.3–92.7); NPV: 90% (95% CI: 54.1–99.5). Areas under the ROC curves for TNF-*α* and MMP-9 were 0.828 and 0.768, respectively.

Exact logistic regression showed that increasing MMP-9 levels were associated with a significant increase in the risk of no response (OR=2.84; 95% CI: 1.11–10.44, *P*=0.024), whereas TNF-α levels did not show such association. A stepwise discriminant function analysis further revealed that MMP-9 levels are better predictors of clinical benefit than TNF-α levels. The discriminant model showed that MMP-9 levels over 4062 ng ml^−1^ significantly (*P*=0.027) predicted sunitinib's lack of clinical benefit (Wilk's *λ*=0.767; *χ*^2^=4.913).

### Baseline levels of MMP-9 and TNF-α are associated with survival

**Figure 3:**
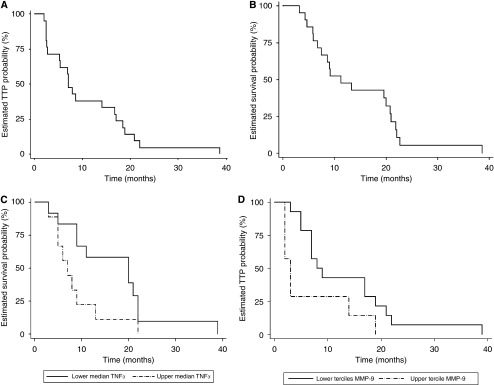
Kaplan–Meier plots of time-to-progression (**A**) and overall survival (**B**) in the complete group. Baseline levels of TNF-α above the median are significantly associated (*P*=0.045) with reduced overall survival (**C**). MMP-9 levels over the upper tercile are significantly associated (*P*=0.042) with decreased time-to-progression (**D**).

Median time-to-progression (TTP) for the whole group of patients was 7.03 months (95% CI: 5.51–8.55) (Figure 3A) and median overall survival (OS) was 11.27 months (95% CI: 4.88–17.65) (Figure 3B). Median OS of patients with TNF-α baseline levels above the median (50 pg ml^−1^) was 7 months (s.d.: 1.491; 95% CI: 4.078–9.92), as compared with 20 months (s.d.: 6.803; 95% CI: 6.65–33.34, *P*=0.045) for the other patients (Figure 3C). A significant association between TNF-α levels higher than 50 pg ml^−1^ and reduced OS was found (*P*=0.045). TTP correlation with baseline TNF-α levels, approached, but did not reach statistical significance (*P*=0.07). For MMP-9, using the median as a cutoff value, no differences were observed. However, levels above the upper tercile (4062 ng ml^−1^) showed a significant association with reduced TTP (*P*=0.042). Patients with MMP-9 levels above that value had a median TTP of 3 months (s.d.: 0.598; 95% CI: 1.83–4.17) as compared with 8 months (s.d.: 1.88; 95% CI: 4.33–11.67) for patients below that limit (Figure 3D). No association was observed between MMP-9 baseline levels and OS (*P*=0.515).

Cox-regression analysis showed a significant interaction between both MMP-9 and TNF-α levels in predicting the risk of reduced TTP: HR for the interaction was 10.323 (95% CI: 1.028–103.64, *P*<0.047), whereas HR for TNF-α alone was 2.63 (95% CI: 0.986–7.058, *P*=0.053) and for MMP-9 alone, 3.05 (95% CI: 1.09–8.51, *P*=0.033).

### MMP-9, SDF-1, and BDNF serum levels are modulated by sunitinib

**Figure 4:**
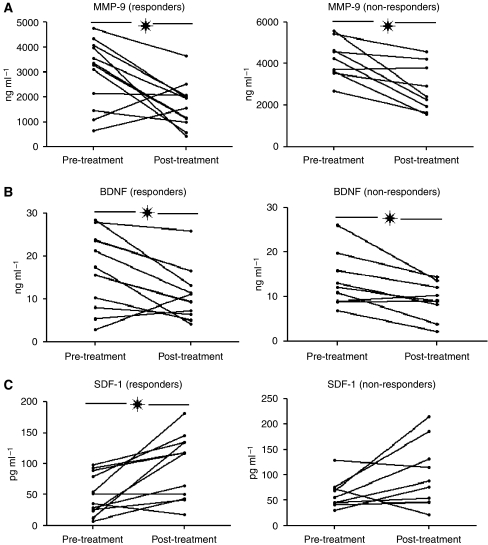
Sunitinib modulates serum levels of MMP-9 (**A**) BDNF (**B**), and SDF-1 (**C**). Both MMP-9 and BDNF levels are reduced by sunitinib, whereas treatment increases SDF-1 levels. ^*^P<0.05.

Baseline levels of the cytokines quantified by ELISA were compared with those measured at the moment of response evaluation. MMP-9, SDF-1, and BDNF levels significantly changed upon treatment (Figure 4), whereas TNF-α, VEGF, and ICAM-1 did not vary. Sunitinib significantly decreased MMP-9 levels in both patient groups (*P*=0.011 for patients with clinical benefit, and *P*=0.005 for patients with progression). BDNF levels also decreased in both groups (*P*=0.016 and *P*=0.011, respectively). On the contrary, SDF-1 levels increased significantly (*P*<0.004) in patients presenting clinical benefit, whereas in patients who progressed, the increment approached statistical significance (*P*=0.08, Figure 4). Therefore, MMP-9, SDF-1, and BDNF seem novel cytokines modulated by sunitinib.

## Discussion

Several new agents are available for treatment of MRCC and many others are under clinical development, leading to a situation in which the choice of the optimal therapy for each patient is sometimes unclear. Predictive markers of clinical benefit are needed to select the most appropriate treatment for each patient. In an attempt to improve the yield of our study, we selected patients with phenotypes of either marked responses or clear progressions and we screened their serum samples with a protein array assessing 174 cytokines. This allowed us to select the cytokines that significantly clustered both groups of patients. Quantitative analysis by ELISA of those cytokines in the entire group of patients confirmed that TNF-α and MMP-9 baseline levels were significantly higher in non-responders. In addition, the area under the ROC curves of TNF-α and MMP-9 indicated good accuracy for both markers as predictive factors of sunitinib clinical benefit. These results suggest that baseline serum levels of TNF-α and MMP-9 warrant further evaluation in future studies as predictive markers of sunitinib activity in patients with MRCC. Our study also supports that our methodological approach seems useful to select candidate predictive factors of clinical benefit of a determined treatment, and that it can reveal alternative pathways that might be relevant to understand the drug's mechanism of action.

Metalloproteinase-9 (MMP-9) belongs to the family of matrix metalloproteases and has a key role in promoting angiogenesis and metastasis (Bergers *et al*, 2000). In the bone marrow, VEGF and SDF-1-mediated activation of MMP-9 releases soluble Kit-ligand, permitting the mobilisation of endothelial and hematopoietic stem cells (Heissig *et al*, 2002). Elevated serum levels of MMP-9 are associated with tumour invasion and metastatic spread in a variety of malignancies. Serum levels and tissue expression of MMP-9 are higher in renal cell carcinoma as compared with healthy controls (Lein *et al*, 2000). Tissue MMP-9 levels in renal cell carcinoma samples are strongly associated with high nuclear grade (Kawata *et al*, 2007) and shortened survival (Kallakury *et al*, 2001).

Pathological regulation of TNF-α has been related to primary and metastatic cancer promotion, directly or via a complex network of cytokines, chemokines, and matrix metalloproteases (Kulbe *et al*, 2007). TNF-α can be secreted by immune-related cells and tumour cells, and it has been suggested to act in an autocrine/paracrine way to promote cancer development (Harrison *et al*, 2007). Both TNF-α and its receptors are coexpressed by renal carcinoma cells and, upon binding, the expression of angiogenesis-related genes such as VEGF (Yoshida *et al*, 1997) and IL-6 has been reported (Ng *et al*, 1994). The potential involvement of TNF-α in renal cancer has been recently demonstrated. High affinity antibodies that target TNF-α, preventing its binding to TNF-αR1 and R2 receptors are clinically available for treatment of rheumatoid arthritis and Crohn's disease. Such antibodies have also been tested as treatment for MRCC at standard and high doses in 37 patients, producing partial responses in three patients (8%) and stabilizations in 11 patients (30%) (Harrison *et al*, 2007). They also reported that baseline TNF-α levels were predictive of clinical benefit. These results along with ours highlight the potential role of TNF-α as a biomarker in MRCC and support the possibility of combining sunitinib with TNF-α blocking therapies.

We have also identified by high-throughput analysis that SDF-1 and BDNF are modulated by sunitinib. SDF-1 is secreted by carcinoma-associated fibroblasts and stimulates cancer cell growth directly through the CXCR4 receptor expressed on tumour cells, and also recruits endothelial progenitor cells into tumours, thereby fostering neoangiogenesis (Orimo and Weinberg, 2006). The SDF-1/CXCR4 pathway has been suggested as a target for renal cancer therapy (Reckamp *et al*, 2008), and dose-dependent increases in SDF-1 levels have been reported previously in normal nontumour-bearing mice treated with sunitinib in a previous study (Ebos *et al*, 2007), which further supports our results. BDNF is a novel angiogenic factor that upregulates MMP-2 and MMP-9 and promotes their activation (Sun *et al*, 2006). Interestingly, anti-BDNF antibodies in multiple myeloma models produce inhibition of tumour growth and angiogenesis (Hu *et al*, 2007). Expression of the BDNF receptor (TrkB) in Wilm's tumours is associated with poor prognosis, probably because it provides autocrine growth-stimulating signals (Eggert *et al*, 2001). BDNF/TrkB should be considered in future studies to assess its potential role as a biomarker and therapeutical target in renal carcinoma.

Other studies have evaluated different potential biomarkers of sunitinib activity. HIF*α* and 2α levels in cell lines and in human tumours have been shown to correlate with clinical response to sunitinib in patients with MRCC (Patel *et al*, 2008). Low baseline levels of sVEGFR-3 and VEGF-C have been associated with a longer TTP and an improved response rate (Rini *et al*, 2008b). Other investigators have reported that patients with gastrointestinal stromal tumours that showed clinical benefit with sunitinib had a reduced number of circulating endothelial cells and monocytes, as compared with patients who did not respond (Norden-Zfoni *et al*, 2007). Greater variations in VEGF, sVEGFR-2, and s-VEGFR-3 were observed in patients presenting objective tumour responses than in patients with stable disease or progression (Deprimo *et al*, 2007). Finally, some clinical factors have been evaluated as predictors of clinical activity. Motzer *et al*, (2008a) have developed a clinical nomogram that predicts the probability of a 12-month TTP in patients treated with sunitinib. Rini *et al* (2008c), have shown that high levels of diastolic blood pressure during treatment with axitinib, a potent and selective inhibitor of VEGFR1, 2 and 3, shows a marked correlation with survival in a series of patients with different tumours. Choueiri *et al* (2007) have reported that five clinical factors, – time-to-diagnosis, baseline calcium and platelet and neutrophil counts and performance status are independent prognostic factors for patients with MRCC treated with anti-VEGF therapy and have developed a prognostic model that categorizes such patients into three prognostic groups. These potential predictive factors of activity, along with those determined in our study should be assessed in prospective trials to provide a more accurate prediction of sunitinib activity.

The main limitations of our study are the lack of standardisation of current tools to determine serum TNF-α and MMP-9 levels as well as the limited sample size. Yet, these limitations are partially overcome by previous reports that also support a role for TNF-α and MMP-9 in MRCC, as reviewed above. In any case, confirmatory studies will be required to corroborate if baseline serum levels of TNF-α and MMP-9 are clinically useful in this setting. If so, the use of these markers might be relevant for further development of sunitinib in other tumour types. Additional research will be required to determine if the predictive value of TNF-α and MMP-9 is specific of sunitinib or if they can also be used to predict activity of other drugs used to treat MRCC.

In conclusion, baseline serum levels of MMP-9 and TNF-α merit further study as predictive markers of sunitinib activity in patients with MRCC. Extreme phenotype selection in combination with high-throughput analysis is a valid method to identify candidates who might prove to be useful predictive factors of activity in cancer treatments.

## Figures and Tables

**Table 1:** Patient characteristics

**Characteristics**	***N* (%)**
*Sex*	
Male	23 (74)
Female	8 (26)
	
*Age*	
Median	58
Range	40-83
	
*Performance status*	
0	20 (64)
1	10 (34)
2	1 (2)
	
*Tumour histology*	
Clear-cell carcinoma	31 (100)
	
*Response to sunitinib*	
Partial response	13 (42)
Stable disease	4 (13)
Progression	14 (45)
	
*Previous treatment lines*	
None	13 (42)
1	11 (35)
2	3 (10)
⩾3	4 (13)
	
*Previous treatments*	
Low-dose cytokines	15 (48)
Bevacizumab	6 (19)
Other	5 (16)

**Table 2:** Statistical comparison of mean baseline cytokine levels quantified by ELISA between patients presenting clinical benefit or progression

**Cytokines**	**Clinical benefit (mean±s.d)**	**Progression (mean±s.d.)**	***P-*value**
**TNF-*α* (pg ml^−1^)**	76.94±194.73	164.07±134.80	**0.009**
**MMP-9 (ng ml^−1^)**	2972.31±1337.31	4227.3±933.73	**0.027**
ICAM-1 (ng ml**^−1^**)	445.7±103.77	482.2±157.99	0.900
VEGF (ng ml**^−1^**)	19.57±17.85	14.27±4.38	0.382
SDF-1 (pg ml**^−1^**)	49.17±32.87	61.86±29.33	0.371
BDNF (ng ml**^−1^**)	16.67±8.64	13.61±6.11	0.378

Bold values are statistically significant.
