# Supplementary material for: Identification of TNF-α and MMP-9 as potential baseline predictive serum markers of sunitinib activity in patients with renal cell carcinoma using a human cytokine array
Source: Br J Cancer. 2009 Nov 10;101(11):1876–83. doi: 10.1038/sj.bjc.6605409 (PMC2788252; doi:10.1038/sj.bjc.6605409)
Supplement: Supplementary Information [file 6605409x1.doc]

**Supplementary online material**

**Appendix 1.**

List of 174 human cytokines analysed with the array (Series 2000, RayBiotech, Norcross, Ga.USA)

Acrp30

Activin A

AgRP

ALCAM

Amphiregulin

Angiogenin

Angiopoietin-2

Axl

B7-1(CD80)

BDNF

bFGF

BLC

BMP-4

BMP-5

BMP-6

BMP-7

b-NGF

BTC

Cardiothopin-1

CCL-28

CD14

CK b 8-1

CNTF

CTACK

CXCL-16

DR6(TNFRSF21)

Dtk

EGF

EGF-R

ENA-78

Endoglin

Eotaxin

Eoxatin-2

Eoxatin-3

ErbB3

E-Selectin

Fas Ligand

Fas/TNFRSF

FGF-4

FGF-6

FGF-7

FGF-9

Fit-3Ligand

Fractalkine

GCP-2

GCSF

GDNF

GITR

GITR-Ligand

GM-CSF

GRO

GRO-alpha

HCC-4

HGF

I-309

ICAM-1

ICAM-2

ICAM-3

IFN-gamma

IGFBP-1

IGFBP-2

IGFBP-3

IGFBP-4

IGFBP-6

IGF-I

IGF-I SR

IGF-II

IL-1 R4/ST2

IL-1 RII

IL-10

IL-10 Rbeta

IL-11

IL-12p40

IL-12p70

IL-13

IL-13 R alpha2

IL-15

IL-16

IL-17

IL-18BPalpha

IL-18Rbeta

IL-1alpha

IL-1beta

IL-1ra

IL-1RI

IL-2

IL-21R

IL-2Rapha

IL-2Rbeta

IL-2Rgamma

IL-3

IL-4

IL-5

IL-5Ralpha

IL-6

IL-6R

IL-7

IL-8

IL-9

IP-10

I-TAC

LAP

Leptin

Leptin R

LIF

LIGHT

L-Selectin

Lymphotactin

MCP-1

MCP-2

MCP-3

MCP-4

M-CSF

M-CSF R

MDC

MIF

MIG

MIP-1alpha

MIP-1beta

MIP-1delta

MIP-3 beta

MIP-3alpha

MMP-1

MMP-13

MMP-3

MMP-9

MPIF-1

MSP-alpha

NAP-2

NGF R

NT-3

NT-4

Oncostatin M

Osteoprotege

PARC

PDGF-AA

PDGF-AB

PDGF-BB

PDGFRalpha

PDGFRbeta

PECAM-1

PIGF

Prolactin

RANTES

SCF

SCF R

SDF-1

SDF-1beta

sgp 130

Siglec-5

sTNF-RI

sTNF-RII

TARC

TECK

TGF-alpha

TGF-beta1

TGF-beta3

TGFbeta2

Thombopoietin

Tie-1

Tie-2

TIMP-1

TIMP-2

TIMP-4

TNF-alpha

TNF-beta

TRAIL R3

TRAIL R4

uPAR

VE-Cadherin

VEGF

VEGF R2

VEGF R3

VEGF-D
